# Shaping Ability of TRUShape and XP-endo Shaper in C-Shaped Canals Using 3D-Printed Replicas: A Micro-CT Study

**DOI:** 10.1055/s-0043-1772568

**Published:** 2023-09-20

**Authors:** Natália Pereira da Silva Falcão, Marilia Fagury Marceliano-Alves, Sandro Junio Oliveira Tavares, Pablo Amoroso-Silva, Aline de Almeida Neves, Luan Ferreira Bastos, Ricardo Tadeu Lopes, Wania Christina Figueiredo Dantas, Michelle Paiva Weydt Galhardi, Eduardo Fagury Videira Marceliano, Cinthya Cristina Gomes

**Affiliations:** 1 Department of Dentistry, Health Institute of Nova Friburgo (PPGO-ISNF), Fluminense Federal University (UFF), Nova Friburgo, RJ, Brazil; 2 Department of Endodontics, Faculty of Dentistry, Iguaçu University (UNIG), Nova Iguaçu, RJ, Brazil; 3 Department of Dentistry, University of Grande Rio (UNIGRANRIO), Duque de Caxias, RJ, Brazil; 4 Department of Pediatric Dentistry and Orthodontics, Rio de Janeiro Federal University, Rio de Janeiro, RJ, Brazil; 5 Department of Nuclear Energy, Rio de Janeiro Federal University, Rio de Janeiro, RJ, Brazil; 6 Department of Dental Reseach, São Leopoldo Mandic College, São Leopoldo, Campinas, São Paulo, Brazil; 7 Department of Dental Materials, Sao Jose University, Rio de Janeiro, Rio de Janeiro, Brazil.; 8 Department of Dentistry, Belem General Hospital, Belém, Pará, Brazil

**Keywords:** dental materials, X-ray microtomography, root canal preparation, TRUShape, XP-endo Shaper

## Abstract

**Objective** This study compared the shaping ability of TRUShape and XP-endo Shaper systems on C-shaped root canals replicas using microcomputed tomography (micro-CT).

**Material and Methods** Thirty three-dimensional replicas based on a mandibular second molar classified as C1 type I C-shaped canal were randomly divided into two groups ( *n* = 15): TRUShape (G.TRU) and XP-endo Shaper (G.XP) and instrumented with each system according to the manufacturer's instructions. Changes in volume and surface and the unprepared area of the root canal were measured by scanning on micro-CT before and after instrumentation.

**Results** The unprepared areas were 39% in the G.TRU and 43% in the G.XP group with no significant difference between them ( *p* > 0.05), but both the tested systems left a high percentage of unprepared root canal walls of C-shaped root canals.

**Conclusion** TRUShape and XP-endo Shaper showed a high rate of unprepared areas with similar results after C-shaped root canals replicas for root canal preparation.

## Introduction

C-shaped root canals are considered challenging anatomies to clean and shape. 1 It is a complex anatomy characterized by root fusion and interconnection of root canals by isthmuses flattening, resembling a “C” letter 2 3 with a radicular groove in which small dentin thickness toward the groove is usually seen. 1 In these type of root canals, the type I classification (merging-type canals) with C1 cross-sectional type (an uninterrupted C with no separation or division between the canals) 4 usually possess large canal volumes and surface areas that will be harder to clean and shape in comparison with the symmetrical and asymmetrical canal classifications. 5

Previous studies have shown that rotary instruments are unable to cut dentin throughout the isthmus region and the internal canal walls when preparing C-shaped root canals. 6 7 The instrument movement is usually concentrated in the canal's central region, leaving the canal extremities unprepared and, consequently, accumulating bacteria and debris. This factor can happen in all teeth, mainly in the apical third, but it gets worse in C-shaped and oval canals, regardless of the instrumentation technique. 8 9 10 Several authors reported that a high percentage of unprepared root canal walls might influence the outcome of endodontic treatment and, consequently, on the development or keeping established apical periodontitis. 11 12

To improve the efficiency of root canal instrumentation, especially in noncircular canal anatomies, flexible instruments with innovative designs and movements have been launched in the market. These alternating movements aim to promote a greater contact area with dentin resulting in a more effective reduction of bacteria. 13 The TRUShape 3D Conforming Files (Dentsply Sirona, Tulsa, Oklahoma, United States) is a full-sequence rotary system that has a design with an S-curve on its longitudinal axis and symmetrical triangular cross-section. According to the manufacturer, 14 its design allows higher contact with the canal walls, allowing an effective cleaning and minimally invasive modeling in flattened canals.

Another rotary system that claims greater efficiency in shaping flattened root canals is the XP-endo Shaper (FKG Dentaire, La Chaux-de-Fonds, Switzerland). This instrument is a single-file system made of nickel-titanium with MaxWire alloy (Martensite-Austenite Electropolishing-Flex, FKG) in a snake-like shape that, when introduced into the root canal at human body temperature, converts the alloy into austenitic phase, resulting in a preparation equivalent to an ISO 30/.04. According to the manufacturer, the high flexibility of the alloy used in its composition reduces the risk of creating cracks in thinner roots. 15

Microcomputed tomography (micro-CT) gained considerable space in endodontic studies, given the reliability of its three-dimensional (3D) images, allowing measurements of postinstrumentation changes in the root canal without destroying the sample. 10 This technology has also allowed to obtain high-resolution 3D-printed tooth replicas 16 which are useful for sample standardization, especially in complex anatomies such as C-shaped canals. 17 Thus, due to the lack of studies using 3D-conforming instruments on C-shaped root canals, this study aimed to compare the shaping ability of TRUShape and XP-endo Shaper systems on C-shaped root canals by analyzing the percentage of unprepared areas left by the instruments, using 3D-printed replicas and a micro-CT evaluation. The null hypothesis tested was that both systems used for endodontic instrumentation have the same preparation ability on C-shaped root canals replicas.

## Materials and Methods

After approval by the ethics committee (CAAE: 76413417.8.0000.5626), a second molar C-shaped, extracted for reasons not related to this study, was used as a model for making 3D-printed replicas.

### 3D-Printed Replicas

After selecting the model specimen of a permanent second mandibular molar with C1 cross-sectional type (an uninterrupted C with no separation or division between the canals) 4 was scanned, using a micro-CT device (Skyscan 1173, Bruker micro - CT, Kontich, Belgium) for morphological analysis and 3D printing. For scanning, the following parameters were used: 40 kV, 150 mA, 7.8 μm pixel size, 2240 × 2240 matrix pixels, 1mm Aluminium filter, 800 ms of exposure, 1° rotation step and 360° rotation around the vertical axis, resulting in a total scan time of 29 minutes. The projections were reconstructed in cross-sections using the NRecon v.1.6.9 software (Bruker microCT, Kontich, Belgium), in which algorithms for artifact correction 10 and beam hardening (50%).

**Fig. 1:**
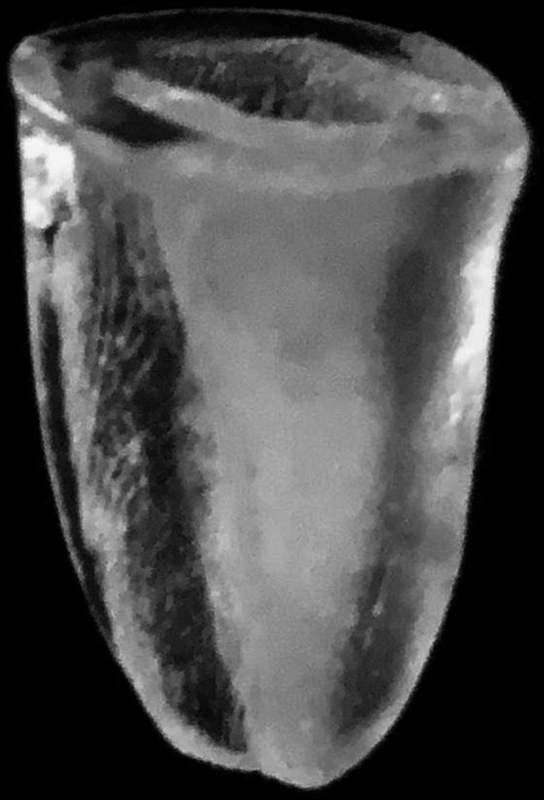
Three-dimensional (3D) replicas printed on resin (VeroClear 810, Stratasys Ltda, Rehovot, Israel).

The cross-sections produced were processed by a sequence of filters using the ImageJ software. 18 19 After filtering, the specimen surface was rendered using the Volume Viewer plugin. The produced map of tetragonal mesh surfaces was exported in *.STL format and imported into the printer software (3D PegasusTouch, 3DGraf, São Paulo, SP, Brazil) where 30 replicas were printed on transparent polymer (VeroClear 810 resin, Stratasys Ltda, Rehovot, Israel) 20 ( Fig. 1 ). Regarding the internal morphological classification, the replicas had type I C-shaped canals according to the 3D classification by Gao et al 21 and continuous C1 cross-section (true C shape and uninterrupted) according to Fan et al. 4

A micro-CT analysis was performed to compare the anatomical similarities through volume and surface area of the replicas in relation to the natural tooth.

### Root Canal Preparation of the Replicas

The canal patency was performed with a K #10 manual file (Dentsply Maillefer, Ballaigues, Switzerland) and confirmed by magnifying the apical region with an operating microscope (MC-M22, DF Vasconcelos, Valença, RJ, Brazil). The working length was established subtracting 1 mm from the patency length and it was set in 18 mm for all samples.

A sample size calculation was performed applying micro-CT results of shaping ability in the formula proposed by Noordzij et al, 22 with an α-type error level of 0.05, a β power of 0.8, resulting in 13 samples (3D-printed replicas) per group. Then, the samples were divided into two groups of 15 teeth each for the instrumentation phase, namely, the TRUShape group (G.TRU) and the XP-endo Shaper group (G.XP).

### TRUShape Group

The TRUShape system was coupled to the X-Smart Plus motor (Dentsply Tulsa Dental Specialties), following the manufacturer's recommendations (300 revolutions per minute [rpm], 3 N.cm) in continuous rotation. The instruments (#20/.06v, #25/.06v, and #30/.06v) were introduced into the root canals with smooth peck movements of approximately 3 mm of amplitude for 10 seconds until reaching the working length. Whenever removed from the canal, the instrument was cleaned with gauze soaked in alcohol. The final diameter was set with the last instrument used as #30/.06v.

### XP-endo Shaper Group

Initially, to keep the XP-endo Shaper (#30/.04) straight for measuring the working length, the plastic tube containing the instrument was cooled with an Endo-Ice spray (Maquira Dental Products Indústria S.A., Maring a, PR, Brazil). The file system was coupled to the X-Smart Plus, following the manufacturer's recommendations (800 rpm, 1 N.cm) in continuous rotation. Pecking movements of approximately 3 mm of depth were performed for 10 seconds up to the working length, which took three cycles to complete canal instrumentation. Whenever removed from the canal, the instrument was cleaned with gauze soaked in alcohol. The final diameter was set in #30/.04.

Instrumentation in all samples was performed with the file insertion in three locations, considering the mesiobuccal, mesiolingual, and distal canals. For the irrigation protocol in both groups, a #30 NaviTip needle (Ultradent, Salt Lake City, Utah, United States) was used, 2 mm below the working length. The canals were irrigated after each instrument insertion with a total volume of 20 mL of 2.5% sodium hypochlorite, 2 mL of 17% ethylenediaminetetraacetic acid for 5 minutes, and 2 mL of saline. A single experienced operator conducted all treatment procedures, and the instruments were used on only one specimen and then discarded.

**Fig. 2:**
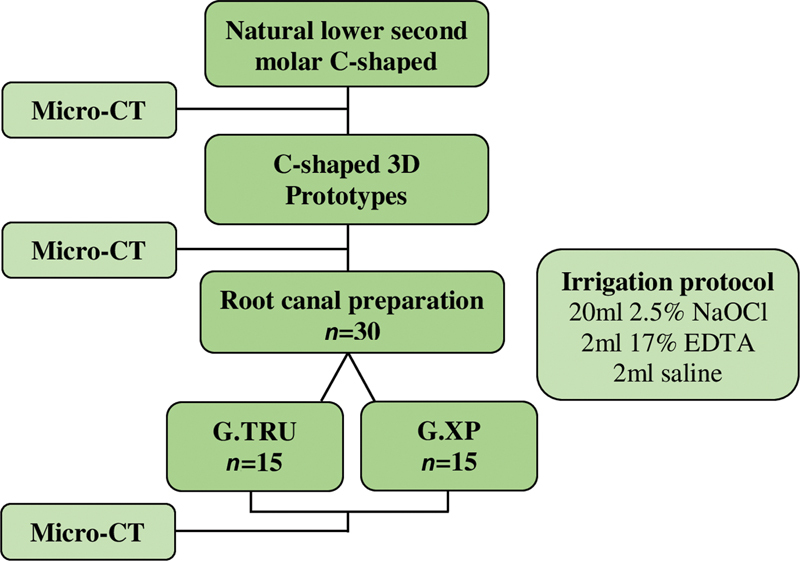
Methodological flowchart.

All the procedures from both groups were performed inside a cabinet at an internal temperature of 37°C maintained by a heater (800-Heater Plas-Labs, Lansing, Michigan, United States) to simulate the clinical environment and allow the martensitic-austenitic transformation from XP-endo Shaper. Then, the specimens were again analyzed using micro-CT ( Fig. 2 ).

### Micro-CT Analysis

The samples were scanned after preparation, and the 3D parameters were assessed by an evaluator blinded to the groups. The analysis was performed on the total length of the root canal replicas before and after the preparation.

The 3D Slicer 4.4.0 software (available at http://www.slicer.org ) was used to register the 3D models of the pre- and postpreparation phases of the samples with a custom combination of rigid recording module based on similarities of image intensity with accuracy greater than 1 voxel. Then, the ImageJ 1.50 days software (National Institutes of Health, Bethesda, Maryland, United States) was used to examine the recorded images and calculate the volume (in mm ^3^ ) and surface (in mm ^2^ ) along the total length of the root canal. The same software was used to evaluate the surface area of the unprepared canal, calculating the number of static voxels (voxels present in the same position on the canal surface before and after instrumentation), expressing the total number of voxels present on the surface of the canal as percentages. 23

### Statistical Analysis

All the morphological parameters evaluated in this study were checked for normality using the D'Agostino and Pearson test, which revealed a normal distribution of the data. The unpaired *t* -test was used for comparison between groups, considering the unprepared root canal walls, canal volume, and surface area changes after instrumentation with the TRUShape system and the XP-endo Shaper. A paired *t* -test was used to compare the volume and surface area increase after instrumentation between the same groups. The mean values and standard deviation and the minimum and maximum values of all data were processed using the Prism 8.0 program (GraphPad Software Inc, La Jolla, California, United States). For all tests, a significance level of 5% was considered.

## Results

**Fig. 3:**
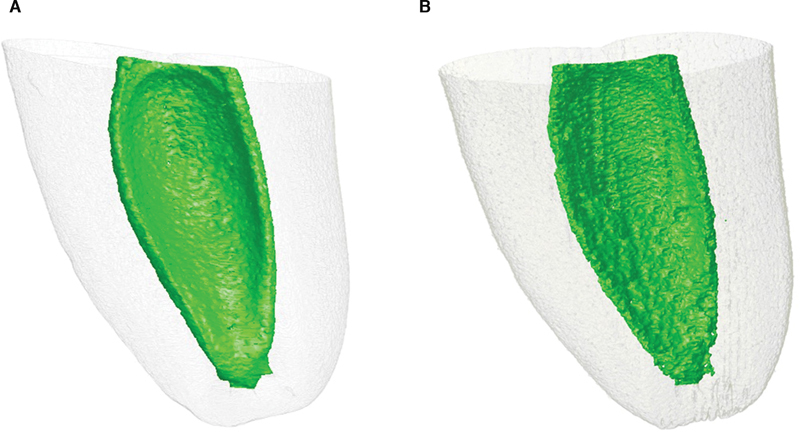
Microcomputed tomography (micro-CT) images. ( **A** ) Natural tooth. ( **B** ) 3D prototype.

**Fig. 4:**
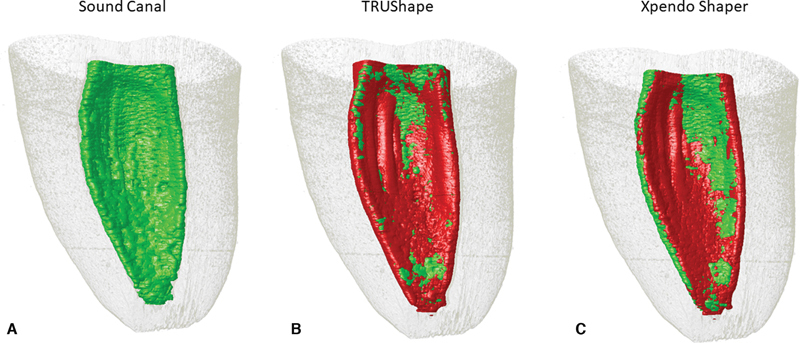
Three-dimensional (3D) reconstructions of the internal anatomy of a C-shaped roots 3D replicas. ( **A** ) Sound molar. ( **B** ) TRUShape system group and ( **C)** XP-endo Shaper system group before (in green) and after (in red) root canal preparation.

No significant difference ( *p* > 0.05) was found in relation to the volume and surface area analysis of the natural tooth compared with the 3D-printed replicas, volume: 19.22 and 18.40 mm ^3^ ; and surface area 176.52 and 172.63 mm ^2^ for natural and printed replicas ( Fig. 3 ). Regarding the root canal instrumentation, volume and surface area increase was observed after preparation with both files system without statistical differences between the groups ( *p* > 0.05) ( Table 1 ). The same was found on intergroup comparison between the initial *and* the final volume and surface area increase at each group ( *p* > 0.05) ( Table 1 ). After root canal preparation, the G.TRU group presented 39% and the G.XP group presented 43% of unprepared canal surfaces. Minimum values found were 7% and 11% and maximum values were 86% and 78% for G.TRU and G.XP, respectively. The unpaired *t* -test showed no statistical differences between both systems ( *p* = 0.3908) ( Table 1 and Fig. 4 ).

**Table 1:** Volume, surface area, and unprepared canal areas of prototype C-shaped mandibular molars before and after instrumentation with both the systems

	Volume (mm ^3^ )	Mean ± SD	Median (min–max)	Surface area (mm ^2^ )	Mean ± SD	Median (min–max)	Unprepared area (%) Mean ± SD	Unprepared area (%) Median (min–max)
G.XP	Initial	18.46 ± 0.60	18.39 (17.03–19.65)	Initial	177.60 ± 2.94	177.90 (170.0–188.30)	43.39 ± 16.27	45.54 (11.18–78.67)
	Final	35.76 ± 6.22	34.47 (24.92–47.81)	Final	260.80 ± 52.18	242.40 (206.40–429.10)		
	% increase	93.83 ± 33.74	84.89 (33.12–161.10)	% increase	46.46 ± 29.90	34.38 (16.21–152.00)		
G.TRU	Initial	18.49 ± 0.72	18.38 (17.22–19.94)	Initial	177.40 ± 1.90	178.00 (170.30–179.80)	38.99 ± 22.66	41.26 (7.71–86.60)
	Final	33.29 ± 7.31	33.23 (20.61–47.81)	Final	254.20 ± 42.23	238.70 (206.40–372.70)		
	% increase	81.01 ± 43.49	78.56 (15.70–166.20)	% increase	43.27 ± 24.10	35.46 (15.93–112.60)		

Abbreviations: G.TRU, TRUShape group; G.XP, XP-endo Shaper group; SD, standard deviation.

Note: There was no significant difference between G.XP and G.TRU in all analyses ( *p* > 0.05).

## Discussion

The null hypothesis was accepted because both systems showed similar results on C-shaped root canals replicas.

In type I C-shaped canals, the C1 canal configuration is most seen at the orifice level. However, this canal configuration changes along the longitudinal direction of the root, and for this reason, these canal cross-section variations might not be predicted in a clinical situation. 4 24 Even though micro-CT studies usually pair-match the group samples based on volume, surface area, and 3D imaging, different canal anatomies and isthmuses shapes are encountered in C-shaped canals, which makes it difficult to acquire paired-matched samples for carrying out *ex vivo* research. Thus, to reduce bias regarding the anatomic variability and its influence on the shaping effects of endodontic instruments or other procedures, previous studies have used simulated resin replicas, 16 25 26 including C-shaped canals. 17 In our study, only one anatomical C-shaped canal prototype was produced to reduce the anatomic variability and its influence on the shaping effects of both tested instruments. Nonetheless, it is worth mentioning that one limitation of studies using 3D-printed replicas is the difference between the dentin hardness and resin replicas, 27 which might affect the shaping ability of endodontic instruments.

One disadvantage of continuous rotary motion instruments when used in complex anatomies is that it tends to centralize preparation, leaving a higher percentage of unprepared root canal walls. 7 8 9 Nowadays, improvements in instrument designs and NiTi alloys created files that rotate eccentrically or asymmetrically inside the root canal. The S-shaped movement of these files contacts more surfaces in noncircular canals. To date, no studies have evaluated the shaping performance of TRUShape and XP-endo Shaper in identical and standardized replicas of C-shaped canals. The TRUShape system consists of four instruments; however, in our study, only three instruments were used. The last instrument used was #30/.0.6 to obtain a more accurate comparison with the XP-endo Shaper, which in the austenitic phase reaches a 0.4 taper, promoting a preparation equivalent to ISO # 30/.04. 15

Previous studies evaluating the shaping ability of TRUShape 10 28 and XP-endo Shaper 29 have shown a considerable reduction of unprepared canal areas in oval-shaped canals. However, in our study, the results still showed a large percentage of unprepared canal areas (TRUShape: 39% and XP-endo Shaper: 43%) in type I C-shaped canals, confirming the proposed null hypothesis. A similar study using micro-CT to assess shaping protocols in type I C1 C-shaped extracted mandibular molars reported different percentages of unprepared areas: 28% after Reciproc and 34% after the self-adjusting file (SAF) instrumentation. 30 Other researchers reported 33% and 30% of unprepared canal areas for Reciproc Blue and XP-endo Shaper, respectively, 7 while higher values of unprepared areas were reported in a previous study using the ProTaper rotary system (66%) and the SAF system (41%). 31 The *in vitro* study of Gazzaneo et al 32 recently reported high disinfecting abilities of XP-endo Shaper, reporting 11.70% of unprepared canal surfaces in C-shaped anatomies. Among these four abovementioned studies, only the study of Amoroso-Silva et al 30 reported the same anatomical classification in the sample selection than our study. Thus, it is evident that the dissimilarity of the results found among these studies might also be a consequence of the sample standardization in such complex and variable internal morphologies of the C-shaped canals.

Although there were no significant differences between groups in the mean values of unprepared canal areas of C-shaped canal replicas, the considerable variation in the minimum and maximum percentages of these result caught our attention: 7.71 to 86.60% G.TRU and 11.18 to 78.67% G.XP. If we consider that all replicas were similar, one might presume that all samples should have yielded similar values. A possible explanation of these different values could be that since the type I C-shape canal has more space for the files to enter in different areas, the noncircular movement of both XP-endo Shaper and TRUShape files could have varied the flute contact of the file against the canal walls from tooth to tooth. Another reason could be that despite that only one operator performed all instrumentation, it is undeniable that pressure or up and down movements applied on the instrument during shaping could differ between procedures.

Regarding the other morphometric parameters evaluated in this study, both systems were able to effectively increase the canal volume and surface area of all samples, which would allow a better flow of the irrigation solution along all the canal surface, resulting in removal of tissue remnants, bacteria, and debris. 33 34 35 36 Nevertheless, the permanence of unprepared canal areas in the root canal could harm the following stages of endodontic treatment. It favors the accumulation of debris into the isthmus recesses during canal instrumentation, which impedes the flow of the filling materials during root canal filling. 35 Possible strategies to overcome these limitations suggested in the literature are complementary instrumentation techniques to reduce the unprepared canal surfaces, 30 36 and the use of passive ultrasonic irrigation or XP-endo Finisher to significantly reduce the accumulation of hard tissue debris, 7 and probably this should be considered as final treatment protocols in C-shaped root canal anatomies.

Regardless of the instrument used, a large percentage of walls remain unprepared. Therefore, new studies must be performed to search for instruments and irrigation protocols capable of promoting greater predictability in preparing C-shaped root canals anatomies.

## Conclusion

Within the limitations of this study, the present results showed that the eccentrically or asymmetrical rotating movement of the TRUShape file system and XP-endo Shaper single file system resulted in a high rate of unprepared canal areas in type I/C1 3D-printed replicas of C-shaped root canals leaving around 38% to 43% of the canal surface unprepared. Both systems significantly increased the canal volume and surface areas without differences between them. Thus, it is important to interpret our results with caution when extrapolating them to a clinical situation.
